# The tip of an iceberg? Adult children’s experiences with parental suicidal behaviour in childhood

**DOI:** 10.1080/17482631.2025.2459299

**Published:** 2025-01-30

**Authors:** Geir Tarje Bruaset, Jennifer Drummond Johansen, Tine K. Grimholt

**Affiliations:** a Centre of Diaconia and Professional Practice, VID Specialized University, Oslo, Norway; b Oslomet University, Oslo, Norway; c Faculty of Health Science, Vid Specialized University, Oslo, Norway

**Keywords:** Parental suicidal behaviour, next of kin, mental illness, children, family

## Abstract

**Purpose:**

This study examined how children exposed to parental suicidal behaviour experienced their parents’ suicide attempts, their relationship with their parents, and family life from childhood to adult life.

**Methods:**

This qualitative, exploratory study conducted semi-structured interviews with 11 participants recruited from next-of-kin organizations and social media. Open-ended interviews addressed childhood experiences, perceived difficulties, and helpful aspects. Interviews lasted approximately 70 minutes and were recorded and transcribed. Thematic analysis was utilized to analyse the data.

**Results:**

We identified two main themes: an unstable, unpredictable upbringing environment and a lifelong responsibility. Participants did not regard their parent’s suicide attempt as the most traumatic event of their childhood. Instead, they identified parental instability due to serious mental illness and/or substance abuse as the most stressful factor. This instability imposed significant emotional and practical responsibilities on the children, forcing them to sacrifice their own development and sense of security. Responsibility was characterized as a life lasting burden.

**Conclusions:**

Adult children of parents with suicidal behaviour reported that their parent’s mental illness was the most burdensome aspect of their upbringing, leading to an unstable home with significant responsibilities. These individuals require professional follow-up and should be offered individual counselling sessions as a clinical intervention.

## Introduction

Suicide is a serious public health problem affecting all countries worldwide. According to the World Health Organization (WHO), there are nearly 800,000 suicides annually worldwide, with suicide being the fourth leading cause of death among 15–29-year-olds (World Health Organization [WHO], 2024). For each suicide, there are approximately 10 times as many attempts. Between 4–6000 people attempt suicide each year in Norway (Folkehelseinstituttet, 2024).

Many patients are hospitalized in mental health care institutions due to suicidal behaviour (Berg et al., 2017). Individuals admitted due to suicidal symptoms often suffer from mental illness. Furthermore, approximately one-third of those who die by suicide have a mental illness (Nasjonalt Senter for Selvmord og Selvmordsforebygging, 2024). Although the reasons for suicide attempts appear complex, affective disorders and previous suicide attempts as well as feelings of hopelessness are well-known risk factors. Additionally, conflicts and breakdowns in important relationships and substance abuse increase suicidal behaviour (Walby & Myhre, 2020). Patients suffering from psychosis and personality disorders have an increased risk of suicide (Snoek & Engedal, 2018). Factors contributing to suicidal behaviour are rarely singular. They are complex and often described by a sense of hopelessness, with death as a possible solution.

Previous qualitative and quantitative research has merely focused on identifying risk factors and demonstrated an increased likelihood of mental illness, physical illness, drug abuse, and suicidal behaviour among offsprings (Goodday et al., 2019; Lunde et al., 2018). Additionally, children exposed to parental suicidal behaviour may have an increased risk of engaging in self-suicidal behaviour themselves in early life stages (Goodday et al., 2019). Cerel et al. (2016) found that nearly one in four children had witnessed their parent’s suicide attempt while one in three knew of it. Children exposed to parents suffering from mental illness are more susceptible to developing emotional difficulties and behavioural disorders (Reupert & Maybery, 2016; Ruud et al., 2015). Furthermore, children exposed to stressors are especially vulnerable (Reupert & Maybery, 2016; Ruud et al., 2015). Without a fundamental understanding of the situation and in the absence of supportive adults, children may be forced to navigate these challenges alone (Reupert & Maybery, 2016; Ruud et al., 2015).

Qualitative research on next of kin after suicide attempts is limited, particularly concerning children exposed to parental suicidal behaviour. Existing studies reported that children found the situation exhausting, were on duty around the clock, and feared repeated parental suicide attempts (McLaughlin et al., 2014), and that growing under such conditions were a burden for them (McLaughlin, 2014; Wenger et al., 2022). They reported a significant fear of losing their parents and felt a profound sense of duty (McLaughlin et al., 2014). People exposed to suicidal behaviour of a close relation were frequently confronted with thoughts of death, which they perceived as traumatic, leading to the development of anxiety and depression (Wenger et al., 2022). Family members experience of caring for people who have attempted suicide is described as impending burnout (Sun & Long, 2008).

## Context of the study

**Figure 2.**
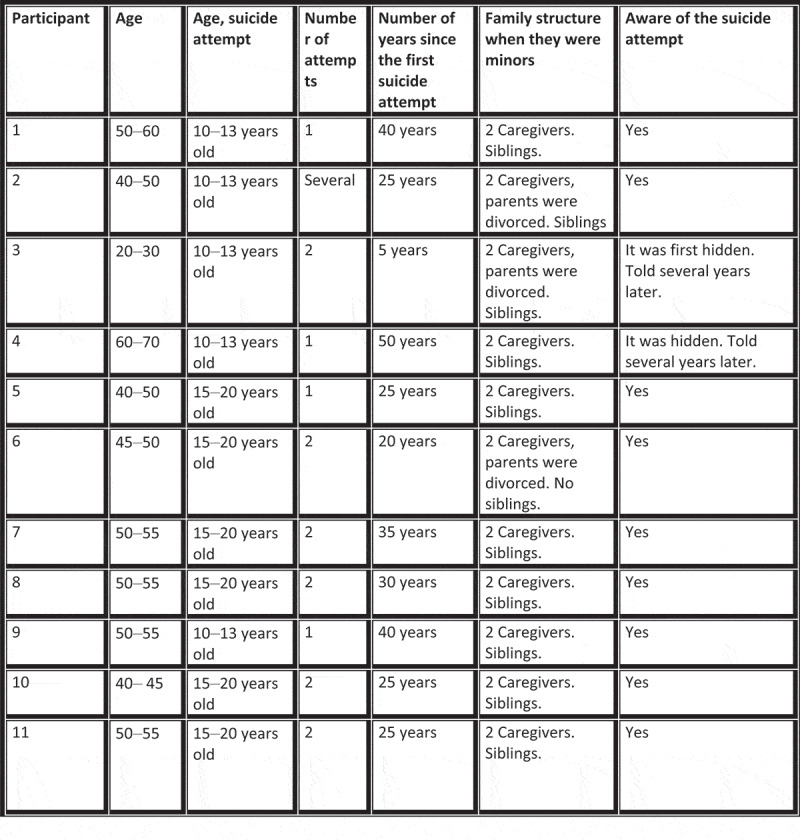
Participants.

This study examined the experiences of Norwegian adults who grew up with a parent who attempted suicide (Refer to Figure 2). Norwegian legislation from 1999 mandated that hospitals care for patients’ relatives, including informing children about hospitalization and treatment (Health Personnel Act, 1999). It also required healthcare professionals to assess the well-being of the children of relatives and provide counselling, provided they have obtained parental consent (Specialist Health Service Act, 1999). There is limited research regarding implementation interventions involving children that have experienced a parent attempting suicide is limited (Lunde et al., 2018). The participants grew up during a time when support for children of parents who attempted suicide was still limited.

Despite knowledge on exposure to traumas, well-documented risk factors, and surrounding upbringing conditions, interventions that include children in parental suicide attempts are limited (Lunde et al., 2018). To provide sufficient help, understanding children’s own experiences and needs during their childhood is essential.

Accordingly, this study aimed to explore how adult children retrospectively experienced living with a parent who attempted suicide and how it affected their relationship from childhood into adulthood.

## Theoretical perspectives

The theoretical framework of our study adopts an environmental, transactional understanding of development (Sameroff, 2010), which reflects the belief that both context and children mutually stimulate each other. Within this perspective, children are acknowledged as active agents in their own lives. Furthermore, developmental trajectories are viewed as a dynamic interplay between the child, caregiver, and overall contextual forces that impact the family (Røn Larsen & Stanek, 2015). Developmental trauma refers to repeated exposure to traumatic events through childhood, often interpersonal in nature, which can lead to developmentally adverse consequences (Gregorowski & Seedat, 2013). This encompassing term refers to a “complex relationship, where children experience traumatic events alongside inadequate caregiving” (Nordanger & Braarud, 2020, pp. 23–24). This term was chosen as it applied to “trauma that occurred during developmentally sensitive periods of life, which interfered with the child’s further development (Briere et al., 2008; Felitti et al., 1998). Developmental trauma entails a complex relationship where such stress occurs in combination with the child not receiving the necessary support to regulate its impact. In this scenario, the child experiences intense distress, and the parent, who should provide reassurance and safety, becomes a source of danger or insecurity (Robinson et al., 2009). Hence, developmental traumatization refers to a situation where this disparity becomes central to a child’s experience (Nordanger & Braarud, 2020).

## Materials and methods

The study is part of a broader research project that aims to comprehend the encounters of offsprings who have matured under the care of a parent with suicidal behaviour and identifying the required support mechanisms for parents hospitalized after a suicide attempt. We aimed to understand participants’ experiences of their situation. Therefore, we chose a qualitative exploratory design with a narrative approach in the interview guide. We obtained data via semi-structured interviews and utilized Braun and Clarke’s thematic analysis to analyse the content.

We recruited 11 adults (nine women and two men) exposed to parental suicidal behaviour in their upbringing via next-of-kin organizations, social media, and the snowball method. No relationships were established prior to study commencement. The first author contacted the next of kin organizations via email and sent an information letter regarding the study. Subsequently, various organizations shared information regarding the study on their websites. Several participants contacted the first author via email. Two participants reached out to the first author after learning about the study through the media or via referrals. We provided participants with the study information and informed consent forms and arranged the time and location for their interviews. Participants were from various parts of the country, resided in larger cities and smaller areas, and had an average age of 50 years.

## Inclusion criteria

The inclusion criteria specified participants who had been exposed to parental suicidal behaviour during their upbringing and were 18 years of age or older.

## Exclusion criteria

Exclusion criteria were individuals affected by serious mental illness and/or those unable to provide informed consent.

## Data collection

Interviews were conducted from May 2023–January 2024. Most took place at the researcher’s workplace. However, for participants who resided far away, interviews were conducted either at their location or digitally. Interviews lasted approximately 70 minutes (range 45–85 minutes) and were recorded and transcribed by the first author. Prior to the interview, the researcher was concerned of the sensitive nature of the topic. Before each interview, participants were informed about the questions that would be asked. They were encouraged to respond freely, take their time to think, and move between themes as they wished. Open-ended questions were posed initially, and participants who were less forthcoming were subsequently asked more direct questions to help facilitate the conversation. Some participants were verbally active and provided rich material while the researcher held a more passive, listening position. Others were more passive, and the researcher held a more verbally active approach. After the interview, participants provided feedback on the setting and context and their observations. They were also provided with material to assist their reflections. They were offered someone to talk after if required. The researcher observed that the participants expressed appreciation for sharing their experiences. After 7–8 interviews were conducted, we found that the quality of dialogue had provided rich material that included detailed narratives, descriptions, and explanations that could offer insights into the complexities of the subject being studied and therefore due to time constraints decided to not recruit more participants. Malterud et al. (2015) refers to this as information power in qualitative studies, wherein the researcher had to weigh the usefulness of including additional participants. No participants withdrew from the study.

## Interview guide

A semi-structured interview guide was developed and designed by all the authors. The first author proposed questions and discussed them with the other authors. The first author received co-authors’ feedback to expand the questions that aimed to elicit participants’ own understanding and experiences throughout their childhood, described in their own words. The guide was narrative-oriented and comprised open-ended questions designed to explore the participant’s understanding of their situation, family relationships (particularly with their parents), and personal history during childhood, adolescence, and transition to adulthood. A pilot interview with an individual with user experience provided important feedback on the importance of not strictly adhering to the guide and instead allowing flexibility of jumping between topics. This was particularly important as some participants shared stories from their childhood that required time to recall details that dated back decades. We initiated the interview by posing the question, “Could you share some details about your daily life?” Subsequently, we asked: “What are your thoughts regarding the topic of growing up with a suicidal parent?” Next, we discussed the childhood home and experiences that the participants had related to the theme. Each interview was concluded by asking whether the participant wanted to add anything that was not discussed. Additionally, owing to the sensitive nature of the topic, participants required time to consider their thoughts and engage in reflection. Consequently, feedback was incorporated into the interviewing process.

## Data analysis

The project aimed to explore participants’ experience via an inductive, narrative approach. We chose reflexive thematic analysis, based on Braun and Clarke’s (2022a). Braun and Clarke’s analysis process comprised six phases: familiarization with the dataset, coding, generation of the initial themes, development and review of the themes, refining, defining, and naming the themes, and write up (Braun & Clarke, 2022b, pp. 6–7).

The first author conducted the interviews and transcribed them. Between the interviews, the co-authors discussed the need for adjustment as well as reflections on positive experiences. Adjustments could be when the interviewer demonstrated patience and allowed the participant sufficient time to answer a question, rather than become inpatient and immediately move to the next question. Positive experiences could be exemplified when the interviewer had a more laid-back role and where the participant spoke freely without necessarily sticking to any sequences.

**Figure 1.**
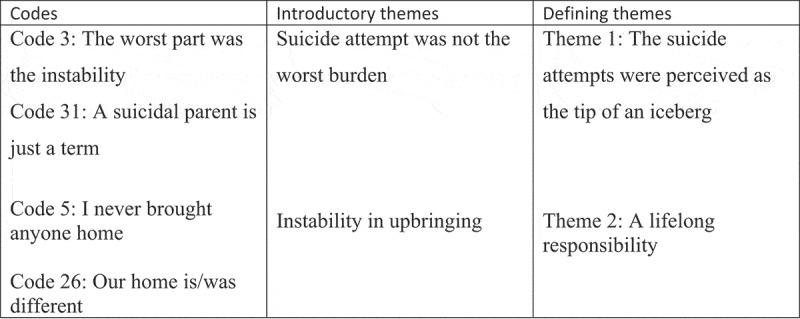
Excerpts from the analysis process.

In the first phase, the first author familiarized themself with the data (refer to Figure 1) and took note of their immediate thoughts and impressions. The second and third phases involved definition of the initial codes and generation of introductory themes, respectively. In the fourth phase, all the authors critically reviewed the topics, data excerpts, and code sets. They examined the findings in relation to previous research on next of kin and drew upon their own professional experience to provide further insights and interpretations. Children may protect their parents and assume emotional responsibility, which was observed in several participants who highlighted that the suicide attempt was not the most challenging aspect of their upbringing. These findings formed the basis of the first theme. They also revealed that having a parent who was severely mentally ill significantly influenced their relationship with their parents, which extended beyond childhood and adolescence into adulthood. Participants described a lifelong sense of responsibility towards their parents that went beyond a typical parent-child relationship. In the fifth phase, the authors collectively defined the themes and sub-themes. Finally, in the sixth phase, quotes that exemplified each theme were selected.

## Reflexivity

Braun and Clarke (2022b, p. 13) suggested that reflexivity went beyond self-reflection and involved an awareness of the knowledge produced through research and methodologies used to acquire it. The first author had experience as a nurse in mental health care and had worked with both patients who had attempted suicide and their children. Furthermore, all authors had extensive experience working with children and adults in vulnerable life situations. This prior experience shaped the approach to the research process and influenced the analytical choices. This study aimed to illuminate the multifaceted experiences of children raised by a suicidal parent and identify ways in which the professional support system, friends, and extended family could effectively assist them. Challenges associated with having an insider perspective included lacking critical distance, advantage was familiarity with the subject, and ability to understand where the shoe pinched. Authors involvement in the analysis work, as well as reflection of their own standpoint, allowed for broader data interpretation.

## Ethical approval

This study was approved by the Norwegian agency for shared services in education and Research (ref nr: 124,333) and Norwegian Regional Ethics Committee (ref nr: 562,376).

All participants signed informed consent forms after they were informed of the study, possibility of withdrawing consent in advance, and an offer of a conversation after the interview. The interview was conducted in a safe environment chosen by the participant. Additionally, they were provided the contact information of a researcher who could be contacted 24/7.

Participants’ names, ages, and backgrounds were anonymized. In cases where age, occupation, or name were provided, this was fabricated to provide a more vivid language and picture of the participant.

## Results

A parent’s suicide attempt was not perceived as the most dramatic experience during participants’ childhood and adolescence. It was identified as the tip of an iceberg, which represented a way out of an existence with instability and mental illness. Our findings underline the relational and emotional impact of parental illness on children and reveal the implications for their environmental upbringing conditions, both in adolescents and adulthood.

## Theme 1: an unstable, unpredictable upbringing environment

A shared theme among participants was that the suicide attempt was not considered the most traumatic incident in their childhood. They described a persistently unstable and dysfunctional environment in which the parent did not have the emotional or practical capacity to take care of the children. One participant shared this story:

**Participant 2**: The hardest part was all the instability around her. So, growing up with a suicidal parent for me is a small part of… The fact that she does not want to live is a small part of it. It is all the other stuff around that has been hard for me. The extreme instability that goes on in cycles.

Another participant reflected on their experience and expressed that, as an adult, she believed witnessing a parent attempting suicide must be a terrible ordeal for a child. However, her own experience did not align with this belief. While it was painful when it occurred, the family managed to move forward. The participant shared:

**Participant 10**: When you think about growing up with a suicidal parent, that is just kind of a term to me. I did not think like that as a child. Yes, he tried to kill himself two or three times. And then - you kind of just moved on. It was not something like that… Because, preparing for this conversation, I have written down that if you look at it from the outside, you might think that it must have been a terribly dark, sad, painful, difficult lifetime. But it was not like that. It was very brutal when it happened. But simultaneously, my mom has been there a lot for me, and my dad was there too, in a way. If I had heard someone else grow up with a suicidal parent, I would have thought, “God, that is terrible. It must have been kind of constantly dark, in a way.” But that is not how I experienced it myself.

Some participants described that they felt guilty as they believed that it would have been easier if the parent had not survived the suicide attempt. This experience was rooted in a sense of not being seen. Furthermore, if the parent had not survived, the children might have been recognized and helped. A participant shared:

**Participant 8**: I have thought and felt guilty that it would have been easier if my mother had managed to fulfil a suicide attempt, because then we would have been helped. As things turned out for us, it became our job to be supportive. We were not seen. At all. It is an ugly thing to say, but that is how it was …. So I remember that as very difficult, it was kind of that guilt because then I had thought she was going to die. So, then it was very hard to realize that she was going to survive, because my dad said that now we had to be very nice and kind, and he was sort of such a strict type and kind of like that to my sister, did not talk to me that much. So, then I felt like there were no other adults there and kind of held around us. And dad probably had a really hard time too… And it was not like my sister, and I could support each other, either. Then we were sort of tasked with helping my mom back to life. That was the role we got.

Some participants described the suicide attempt as not unexpected; instead, they understood why. They perceived it as a desperate turning point after many years of despair and instability. One participant shared:

**Participant 9**: And we realized, we had no problem understanding why she was trying to kill herself. There was an awful lot of noise at home, they were fighting too, she was trying to kill dad. He defended himself, there were bruises and stuff like that, so we had bedrooms, or I had bedroom right next to them, and every single night, I have a brother and sister, they would come into me and lay down next to me. So, me being the oldest, then, I think that I became such a security for them.

Several participants described significant responsibilities as children, which included completing practical tasks that their parents were too exhausted to perform. However, this also included providing emotional support to their parents, often at the expense of their own feelings and thoughts. Additionally, their responsibilities included exposure to frightening visuals and scenarios that they stated children should not witness. In some instances, participants discovered their parent after an overdose of medication and quickly called for help. In one case, an 11-year-old boy closely monitored his mother’s Valium boxes to track her intake. On a particular day, he found his mother lying languid on the couch, struggling to breathe. He recounted:

**Participant 2**: I called the ambulance, when I found her on the couch and barely breathing, she had this kind of Valium glass that I knew where she was hiding, so I figured she had taken a lot of those, because I had a record of her pills. A brown glass. She had taken a great many of them. I try to think about how old I was then. Around 11–12 years old…? Then I called them, and they called an ambulance. Then she was sent to the hospital, was probably pumped, and was there for a night or two. Then she came back. By then she was tired and slept a lot.

Another participant recounted her initial meeting with her father after he had been hospitalized following a suicide attempt. Although she had discussed the situation with her mother, no one had prepared her for the sight that awaited her inside her father’s hospital room. While healthcare professionals had provided some limited information about the incident, there was no preparation for seeing her father in that condition. She shared:

**Participant 3**: I visited him in the hospital the next day. Or a few days after. Because then they called me from the hospital to tell me and at the same time, I was given the opportunity to ask any questions that I might have. So, I went there and visited him and then I met a nurse. She told a little, but not much. I was very shocked when I saw he had bruises all over his head and a wound and was bleeding. He was completely yellow. Because they had crammed him full of fluid. And no one prepared me for that. So, I probably looked like I had seen a ghost. I found it very disturbing.

A participant recalled a significant event from adolescence that made a lasting impression. It was an afternoon when the family was watching television, and her dad was at work. Suddenly, her mother started hyperventilating, which caught the participant off guard. The sudden and unexpected episode scared her, and she was not mentally prepared. This event left a lasting impact on her, which also emphasized the challenges the family faced during that period. She shared:

**Participant 7**: It was my mom’s first anxiety attack when we watched Children’s TV. A creepy figure appeared on the screen, and Mom is lying on the couch saying, “That is how nasty it is…”. And that anxiety attack, with that howling, hyperventilation and shaking, I remember thinking, “This is what they are afraid of”… Then I remember my brother coming and saying, “Mari, you need to come and calm her down.” By then, I was 16. I just remember thinking: “Calm her down? I do not know how, I cannot, I have no idea how to calm someone down who behaves like that.” Except to say, “Go to bed. (Laughs a little). Get to bed.” Then she was admitted to an acute psychiatric ward and was there for a week.

At times, events occurred that involved content that children ideally should not witness. A participant vividly described a mother who exhibited highly unstable traits. His mother relied heavily on prescription drugs and consumed excessive alcohol, particularly during weekends. The household was occasionally visited by different men. That made him uncomfortable. He shared:

**Participant 2**: She did not have any good, solid friends. She lost everything; she did not have any capacity for work. There was a lot of drunkenness, in the weekends, especially. From Fridays to Sundays. Mostly. There were men there. I heard people having sex. Girlfriends could have sex in bunk beds under me. By then, my older brother had moved out, so it was just me and my little sister. She was just a child, so there has been a lot of stuff like that. Things kids should not see. I remember one time they turned on the light in the bed below me, the man was wet in the crotch, with his panties… I did not know what an orgasm was at that time. Did not understand things like that. So, it was completely distant then. Sit and watch it. Lots of loud music. I would visit the bathroom, and there was a strange man sitting there.

## Theme 2: a lifelong responsibility

### Immediate responsibility

Participants said that the protectiveness they felt towards their parents sometimes involved being quiet when their parent was tired. However, children’s obedience also included outsiders or professional support system when they were concerned regarding their situation at home, which sometimes came at the expense of their own needs for care and security. Participants described feeling a long-standing sense of responsibility towards their parents, which persisted long after they had left home and even after the death of their parents. A participant shared:

**Participant 2**: But I had once, in connection with my stepfather tried to take over the care, because then a report of concern was probably sent from the Child Protection Services. Then I remember my mom driving downtown, she was nervous for sure… Then she was very cheerful. Sort of tried to say something about what I should say… who I was going to meet. I did not know what I was getting into. And then I remembered talking to some women, but then nothing ever happened. You protect your parents, right? Was not so grilled or drilled that much… Do not know how they worked at the time… But at least I said that everything was fine at home, and there was nothing to worry about… stuff like that. I, like, protected mom then.

Some participants took on multiple practical tasks at home and had little to no time for anything else. A participant reflected that she had few relationships with peers from childhood. Eventually, she became lonely at school and had limited or no friends. She shared:

**Participant 8**: One gives up their own development to take care of their parents. I recognize that. So, it turned out that I did not have a lot of friends eventually, then. Unfortunately. So, my parents never got any help… They sort of never asked… I became the one who did the chores and cooked dinner from the age of 13. We had “food and health” at school, so I used that knowledge to make dinner. Because then my mom started working, she could not bear to do much when she got home. And, then I had to, or felt like I had to contribute. There was sort of no question that “now you do a lot at home, maybe you should get some help to be with your peers.” No one saw that part of it, then.

Some participants highlighted the importance of prioritizing what they believed was best for their parents. They felt that if they were just quiet and did what they were told, perhaps their parents would have felt better. A female participant said this led her to suppressing her own feelings and not expressing them. She shared:

**Participant 9**: We were very loyal to both of our parents, all the time. My mom had bipolar disorder and was manic a lot. After the manic periods, she was very depressed and that was just the way it was, sort of … I remember we did everything we could to make Mom and Dad happy. My sister was the one who got out her emotions more, cried when she was angry. But I was very quiet and tried to be clever enough and stuff. And my brother did not understand much. But we, I think both me and my sister did what we could to make sure both mom and dad were okay.

### Long term responsibility

Participants described a lifelong responsibility to their parents, even after their parents had passed away. However, this responsibility extended beyond a natural sense of responsibility between generations. In their adulthood, participants struggled with feelings of betrayal and an inability to acknowledge the strain they experienced in their childhood. A participant expressed frustration and defeat when he thought of the ongoing responsibility he felt and continued to feel towards his parents.

**Participant 1**: With my father and mother, I was very worried as the eldest son, my brother was not very much present for them… Are they going to die in a pool of blood now? Are they going to… mother run into a tree? And my father… He drank extremely much at the end. Am I just going to find him dead in the kitchen at home? So, there was a lot of responsibility until the very end. A great responsibility, and I must do my utmost to ensure that they have a dignified death. But d**n it! It should not be my responsibility! No way! They need to sharpen up. There will be others who take that responsibility! I cannot take on all the roles all the time for them. And being there, being a cleaner, and then talking about their problems and then arranging finances… D**n it, that is… There will be limits.

Participants also tried to express to their parents how they experienced their childhood while their parents still were alive. However, some felt guilty while others were unable to have this conversation calmly, which ended in a verbal outburst. A female participant, who recently lost her father to natural causes, found a letter while cleaning up her childhood home. It had stamps on it, however, it was never mailed. She shared:

**Participant 8**: I had such an outburst when I was 22–23 years old. And then I said things that maybe were not so pretty… Then I felt very guilty afterwards. I felt guilty for saying those things to my mom. But in retrospect, when I cleaned up after my parents, I found a letter from my father, which he did not send, but wrote to me. Where it said he understood that I was so mad at my mom… He kind of agreed with what I said, then … He then admitted that he had not been able to see us when my mum had tried to attempt suicide, and he wrote that he should have talked to me and my sister. But he did not make it. Because Mom absorbed everything. He had no energy to see us. So, it was kind of a very nice letter he wrote, but I got it 30 years too late. Because it would have made a pretty big difference if I had gotten it in my early 20s. Because then I might have been able to make some distance. Not taken so much responsibility anymore… And now he is gone.

## Discussion

Our findings revealed that most participants did not experience their parent’s suicide attempt as the most traumatic event in their childhood. It was perceived as the tip of an iceberg, which comprised instability, and included exposure to serious parental mental illness, and substance abuse. These factors characterized and influenced the participants’ everyday lives. Additionally, participants perceived a distinct sense of responsibility towards the parents that went beyond a normative parent-child relationship. They undertook responsibilities for both their siblings and assumed emotional burdens for the parents, which ultimately impacted their own development and sense of security.

## Procedural upbringing conditions of children exposed to parental suicidal behaviour

These findings provide insight into the everyday lives of the participants. The children experienced traumatic events individually, alongside general neglect and high levels of stress. Moreover, they lacked essential support from both their extended family and professional support systems. In an increasingly tense environment, participants found themselves waiting for something to happen. They recognized that something was wrong, but it was not explicitly acknowledged in their daily lives. This could explain why they did not perceive the suicide attempt as the most traumatic event; rather, it confirmed that something was indeed not right. Individuals who have experienced violence in close relationships often report similar circumstances. People who experienced violence in close relationship frequently reported similar circumstances (Etzold, 2022). In 1979, American psychologist Walker developed “the cycle of abuse” after interviews with 1500 women and identification of shared experiences in their stories (Etzold, 2022). This cycle typically comprised four stages: tension building, acute violence, reconciliation, and calm. Parallels could be drawn between these stages and the results of this study. Children perceived a sense of unease and tension in their environment, which manifested in symptoms such as panic attacks, substance abuse, or aggression. This tension eventually culminated in the parent’s suicide attempts, as observed in our study. Following these attempts, the parent typically received assistance through hospitalization. Subsequently, efforts were made to reconcile the parent and children. This cycle repeated throughout their childhood, with several participants describing how their parents’ instability followed a cyclical pattern. Participants found this instability particularly stressful, feeling responsible for maintaining as much stability as possible in their environment to avoid further tension.

Literature on children as next-of-kin to parents with severe mental illness indicated that the children in our study shared similar experiences with those of previous studies (Wangensteen et al., 2019; Wiegand-Grefe et al., 2019). Children felt that their parents’ illness dominated their lives, even after their parents’ deaths, and they struggled with conflicting emotions. As adults, they often found it challenging to organize their daily routines (Wangensteen et al., 2019).

Not all children who took on practical and emotional responsibilities when their parents were affected by serious illness experienced these roles negatively. Some reported maturation and growth as a result of assuming early responsibility. However, such experiences could also impact their personal development and hinder them from fulfilling their own needs (Hooper 2017 & Levesque, 2017). Growth may depend on the capability of the adults and parent to recognize that children exposed to certain responsibilities did not necessarily have the prerequisite to understand them (Jurkovic, 1997; Schier, 2014).

Previous research on children affected by parental mental illness revealed that they took on considerably more care tasks and housework than usual (Ruud et al., 2015, pp. 10–13). They also had poorer physical and mental health than other comparable groups (Lacey et al., 2022). Furthermore, they reported a lower quality of life than the general population (Lacey et al., 2022). Parents believed that the children’s quality of life was better than what the children expressed themselves (Ruud et al., 2015, pp. 10–13). Lack of protection from one’s own parents could have a major impact on people later in life, both physically (Nevriana et al., 2020) and psychologically (Patrick et al., 2019). Children who grew up with a parent with severe mental illness often contemplated their approach to parenthood as adults. They prioritized being open and transparent with their own children owing to this crucial aspect (Patrick et al., 2019).

Some participants said that their childhood experiences still bothered them regarding guilt and psychological distress, for which they still received psychiatric treatment. These findings were supported by prior research where the experience from childhood indicated that participants struggled to be parents themselves (Patrick et al., 2019). Parents did not understand their children’s burden when one parent was seriously mentally ill (Ruud et al., 2015). Our findings indicated that the childhood responsibility was burdensome and carried into adulthood. However, some participants were more aware of the importance of being open with their own children regarding taboo topics.

## Severe childhood experiences and developmental trauma

Theories of developmental trauma were central to this study as they defined a dual burden that largely characterized the findings: lack of predictability, information, and emotional support. Additionally, the closest caregivers represented the unpredictability of participants’ daily lives. This double burden relationship was understood as experiences beyond ordinary everyday stressors as well as lack of safe caregivers in everyday life (Nordanger & Braarud, 2020 , pp. 23–24; Robinson et al., 2009). The healthy parent was usually at work, while the children were exposed to the other parent’s mental illness. Five participants reported that the parent who was ill would stay at home while the other parent working outside the home and assumed the practical and financial responsibilities for the family. Consequently, participants handled and dealt with difficult matters largely on their own. This finding indicated that the entire family was affected by a parent’s serious mental illness and suicidal behaviour. It further highlighted the need for professional support services for both the exposed children and their parents.

Our preconception at the start of this study was that the suicide attempt must have been an enormous burden. A participant explicitly stated that if she had heard of someone experiencing a parent’s suicide attempt, she would have expected it to be a terrible ordeal. However, this was not the case for her own self. This was consistent with the experiences of other participants as they also reported it was not the greatest trauma in their upbringing. Conversely, some participants said it would be easier if the parent had fulfilled the suicide attempt. They thought that if it that happened, they would have been seen by both the extended family and professional support service. This finding is important. It highlights the strain experienced, and consequently, the importance of knowledge, follow up, and conversations with children regarding parental suicidal behaviour and serious mental illness. This finding may be connected with prior studies where healthcare professionals were reluctant to include next of kin when parents were admitted to hospitals due to suicidal behaviour. Consequently, children often did not receive essential information and support required to navigate their parents’ situation (Moe et al., 2022). Prior studies suggested that children exposed to parental mental illness were mostly left alone to navigate through their distressing emotions, lack of information, feelings of guilt, and weight of responsibility (Cudjoe & Chiu, 2020).

Several participants reported a cycle of repeated suicide attempts, hospitalizations, and instability. A participant spoke of the shock that greeted her when she visited her father after a suicide attempt. She was not mentally prepared for this vision and reported having limited telephone conversations with hospital staff. Subsequently, all professional follow-up stopped. Prior research revealed that healthcare professionals were not aware of routines for follow-up for children next of kin in mental health care (Hjelmseth & Aune, 2018). A revision of the Norwegian next of kin guide (2021) reported several weaknesses in the follow-up. Management support was identified as a clear improvement point. When parents were hospitalized, healthcare professionals must initiate family-oriented interventions. Furthermore, these interventions must be anchored in leadership (Osloeconomics, 2021).

## Implications

Regarding clinical implications, this study suggests the need for family-centred interventions when a parent attempts suicide. Furthermore, special attention should be provided from healthcare professionals to children’s perspectives and how they perceive their situation. Children should be offered counselling with healthcare professionals alone when a parent is suicidal. When parents are present, children may not openly express their genuine perceptions of the situation to protect one or both parents. Hospital management should have distinct expectations that a key part of working with parents admitted to the hospital involves family-oriented procedures. We suggest future intervention studies that assess conversation-based interventions with children when parents are hospitalized after a suicide attempt. Additionally, further research should explore how themes related to parental suicidality are addressed within the family.

Academic literature defines “resilience” in various ways. According to Lepore et al. (2014), resilience was “an individual’s ability to cope relatively well in situations of adversity,” which highlighted an individual’s ability to manage during difficult circumstances. Similarly, Ungar (2011) defined resilience as “the ability to navigate individually and through social and cultural interactions, despite being exposed to significant adversity.” Children may react differently to adverse events, and perceived support is a crucial preventative factor (Nordanger & Braarud, 2020). Hence, implementation of early interventions can prevent long-term harm and illness (Lunde et al., 2018).

Our study focused on the adverse experiences the participants faced during their childhood and challenges they encountered. Further research on the protective factors that can benefit children that grow up with a suicidal parent may provide a further nuanced picture and improve additional knowledge.

## Strength and limitations

### Transferability

First, this study limited participants to a certain age range. Inclusion of younger participants could have contributed to further nuanced findings owing to recent social trends towards greater openness regarding taboo topics. However, our youngest participants reported similar experiences to those of older participants to a large extent. Regarding the theme of inadequate professional follow-up, the youngest and oldest participant shared identical experiences. Furthermore, both described lack of communication regarding how this impacted their family as extremely burdensome. Our participants experienced the suicide attempt of their parent 20–30 years ago. This likely influenced their interpretation and understanding of the event today. During the interviews, several participants mentioned that they had received therapy to process their childhood experiences, which indicated that their memories may have been influenced by subsequent interventions. However, this also strengthened the study as they provided further verbal and matured reflections.

### Confirmability

We analysed this study according to Braun and Clarke (2022a, 2022b). All authors were involved in the analysis. The first author conducted the initial coding and created preliminary themes, which were developed and finalized via discussion with the co-authors.

To increase the confirmability of our analysis (Braun & Clarke, 2022a, 2022b), we implemented a few measures throughout the process. First, we maintained a comprehensive inspection trail that documented our coding decisions and creation of the themes, which provided transparency and accountability (Lincoln & Guba, 1985). Second, we conducted a check via members and presented our findings to select participants and sought their feedback to verify that our interpretations accurately aligned with their experiences. Finally, we discussed our findings and interpretations with internal and external experts across diverse research settings to ensure that our conclusions were robust and informed (Patton, 2015).

### Credibility

The first author had experience working with patients and their families in psychiatric wards, which may have influenced the results. This was perceived as a strength as it offered a professional perspective. However, this preconception may have led to certain limitations in the research methodology, particularly during the interview process. Participations may have presumed that their responses were already comprehended, which could have led to a lack of elaboration in their statements (Creswell, 2014). We approached this study with a pre-understanding of the topic that may have impacted the interview situation and interpretation of the results. Meanwhile, co-authors without similar experience as the first author may have provided a critical distance. Despite this, our findings were deemed reliable considering their methodology and theoretical perspectives. Previous research on children who grew up with a parent with severe mental illness and substance abuse largely supported several of our findings (Cudjoe & Chiu, 2020; Ruud et al., 2015; Wangensteen et al., 2019; Wiegand-Grefe et al., 2019).

## Conclusion

Parental suicide attempts were not considered as a major trauma in the participants upbringing. Rather, participants attributed the attempt to their parents’ long-standing mental illness and substance abuse. Participants described their parents’ mental illness as persistent and all encompassing, which resulted in a childhood marked by unpredictability, responsibility, anxiety, and neglect of their own needs. They also reported lack of support from both their extended family and professional support systems. Previous research on children with a parent with severe mental illness reinforced our conclusions. Children who grew up with a suicidal parent appeared to confront an extra taboo concerning suicidal issues in addition to mental illness. Moreover, this topic was culturally and socially taboo among healthcare professionals and extended families.
